# A Dual Tracer ^18^F-FCH/^18^F-FDG PET Imaging of an Orthotopic Brain Tumor Xenograft Model

**DOI:** 10.1371/journal.pone.0148123

**Published:** 2016-02-04

**Authors:** Yilong Fu, Lai-Chun Ong, Sudhir H. Ranganath, Lin Zheng, Irene Kee, Wenbo Zhan, Sidney Yu, Pierce K. H. Chow, Chi-Hwa Wang

**Affiliations:** 1 Department of Chemical and Biomolecular Engineering, National University of Singapore, 4 Engineering Drive 4, Singapore 117585, Singapore; 2 SingHealth Experimental Medicine Center, Singapore General Hospital, Block 9, Level 3, Outram Road, Singapore 169608, Singapore; 3 NUS Environmental Research Institute, National University of Singapore, 1 Create Way, Create Tower #15–02, Singapore 138602, Singapore; 4 National Cancer Centre, 11 Hospital Drive, Singapore 169610, Singapore; 5 Office of Clinical Sciences, Duke-NUS Graduate Medical School, Singapore, 169857, Singapore; 6 Department of Chemical Engineering, Siddaganga Institute of Technology, B.H. Road, Tumkur-572103, India; Ohio State University, UNITED STATES

## Abstract

Early diagnosis of low grade glioma has been a challenge to clinicians. Positron Emission Tomography (PET) using ^18^F-FDG as a radio-tracer has limited utility in this area because of the high background in normal brain tissue. Other radiotracers such as ^18^F-Fluorocholine (^18^F-FCH) could provide better contrast between tumor and normal brain tissue but with high incidence of false positives. In this study, the potential application of a dual tracer ^18^F-FCH/^18^F-FDG-PET is investigated in order to improve the sensitivity of PET imaging for low grade glioma diagnosis based on a mouse orthotopic xenograft model. BALB/c nude mice with and without orthotopic glioma xenografts from U87 MG-luc2 glioma cell line are used for the study. The animals are subjected to ^18^F-FCH and ^18^F-FDG PET imaging, and images acquired from two separate scans are superimposed for analysis. The ^18^F-FCH counts are subtracted from the merged images to identify the tumor. Micro-CT, bioluminescence imaging (BLI), histology and measurement of the tumor diameter are also conducted for comparison. Results show that there is a significant contrast in ^18^F-FCH uptake between tumor and normal brain tissue (2.65 ± 0.98), but with a high false positive rate of 28.6%. The difficulty of identifying the tumor by ^18^F-FDG only is also proved in this study. All the tumors can be detected based on the dual tracer technique of ^18^F-FCH/ ^18^F-FDG-PET imaging in this study, while the false-positive caused by ^18^F-FCH can be eliminated. Dual tracer ^18^F-FCH/^18^F-FDG PET imaging has the potential to improve the visualization of low grade glioma. ^18^F-FCH delineates tumor areas and the tumor can be identified by subtracting the ^18^F-FCH counts. The sensitivity was over 95%. Further studies are required to evaluate the possibility of applying this technique in clinical trials.

## Introduction

Gliomas (glioblastoma multiforme, GBM) are the most common primary tumors of the cerebral hemisphere. They are highly malignant and possess a poor prognosis. Similar to all other cancers, the key to survival for glioma patients is early diagnosis. Unfortunately, the early symptoms of gliomas are highly non-specific and patients are usually diagnosed at the late stage of this disease.

Currently, the diagnosis of glioma mainly relies on CT and MRI. Although CT and MR can provide high resolution images, the tumor diagnosis can be delayed since these two imaging techniques rely on the detection of anatomical changes resulted by tumor growth. Contrastingly, PET imaging detects biochemical abnormalities that precedes anatomical changes, and hence may provide an earlier diagnosis of glioma, particularly low grade gliomas. However, the appropriate radiotracer for PET imaging of glioma has not been determined yet.

As the most commonly used PET radiopharmaceutical, ^18^F-fluorodeoxyglucose (^18^F-FDG) is a glucose analogue and is taken up significantly by normal brain cells relative to glioma, making the use of PET in the diagnosis of glioma inefficient and inconclusive except in cases of advanced stage glioma [1–5].

^18^F-Fluorocholine (^18^F-FCH), a choline analogue, is a radiotracer developed for the imaging of prostate cancer. Choline is a precursor for phospholipids and is incorporated into phosphatidylcholine, a primary component for cell membrane construction through the activity of choline kinase. Tumor cells often present an elevated level of choline kinase, resulting in an increased uptake of ^18^F-FCH [6–8]. Nuclear Magnetic Spectroscopy data revealed a high choline content in gliomas [9]. Animal studies showed that ^18^F-FCH is also taken up by glioma with a high tumor to background ratio [10]. Clinical experiments represented that ^18^F-FCH accumulates significantly in high grade glioma [11], but little is known about its distribution into low grade gliomas. Recent clinical studies demonstrated a high sensitivity of ^18^F-FCH PET/CT detection for low-grade glioma based on experiments involving 18 patients [12], with no false positive being observed. However, it still has been reported that ^18^F-FCH can accumulate in normal brain cells [6, 10] to result in false positive.

In this study, the possibility of using dual tracer of ^18^F-FCH/^18^F-FDG for PET imaging in low grade glioma diagnosis is investigated. The main assumption is: ^18^F-FCH may effectively demarcate the glioma from normal cerebral tissues and the presence of tumor may be confirmed on the merged ^18^F-FCH/^18^F-FDG images. The basic principle of the dual tracer technique in this study is owing to the potential false-positive introduced by possible accumulation of ^18^F-FDG and ^18^F-FCH in normal brain cell in the low grade glioma diagnosis with PET imaging applied, the overlapping on the merged ^18^F-FDG/^18^F-FCH PET images is able to effectively identify the tumor and hence improve the diagnosis accuracy.

As indicated in this article, a CT scan is obtained as a reference to determine the brain regions in the ^18^F-FDG and ^18^F-FCH PET images. Since ^18^F-FDG scan may falsely label the region of the normal tissue as the tumor, and ^18^F-FCH scan is able to provide an improved tumor delineation, a tumor Region of Interest (ROI) is firstly drawn manually on the ^18^F-FCH image. The ^18^F-FDG and the ^18^F-FCH images are then merged. The tumor ROI from the ^18^F-FCH can be transferred onto the merged images and the ^18^F-FCH counts are eventually subtracted from the merged images to obtain the finial tumor ^18^F-FDG counts.

## Materials and Methods

### Animal preparation

All animal experiments were approved by the SingHealth Institutional Animal Care and Use Committee (Approved: IACUC #2007/SHS/305 and IACUC #2007/SHS/305A) and carried out in an AAALAC-accredited facility. Five weeks old BALB/c nude mice were used for the study, as 14 mice in the normal control group and 10 mice in the tumor bearing group, respectively. The animals were provided with sterilized food and water *ad libitum*, and housed in negative pressure isolators.

### Generation of intracranial glioblastoma in mice

U87 MG-luc2, a human glioblastoma cell line was purchased from Caliper Life Sciences, Hopkinton, MA. The cells were grown in Minimum Essential Medium (Invitrogen) with 10% fetal bovine serum (Gibco, Invitrogen) and 1% penicillin-streptomycin (Gibco, Invitrogen) in humidified atmosphere containing 5% CO_2_ at 37°C. Cells growing exponentially *in vitro* were trypsinized and harvested for tumor inoculation. All animal manipulations were performed under sterile conditions. In order to obtain intracranial glioblastoma bearing mice, BALB/c nude mice were anaesthetized with intraperitoneal injection of a ketamine/diazepam solution (50 mg/kg ketamine and 5 mg/kg diazepam) and immobilized on a stereotactic head frame (Thermo Fisher Scientific). For subcutaneous administration of baytril, a total dose of 5 mg/kg was injection before operative procedure. Briefly, a midline incision was made on the scalp and a burr hole was drilled 2 mm lateral to the sagittal sinus at the midpoint between the bregma and lambda, and 300,000 U87-MG-luc2 cells suspended in 10 μL of MEM were injected into the hole to a depth of ~ 2.5 mm by using a hypodermic needle (Hamilton Co., Reno, NV). The incision was sutured closed with 5/0 prolene. Postoperatively, the mice were given a subcutaneous injection of 5 mg/kg caprofen. PET scan was performed on the mice one week after the tumor inoculation.

### Micro-PET imaging by using dual tracers of ^18^F-FCH/^18^F-FDG

Each mouse was subjected to ^18^F-FCH scan followed by ^18^F-FDG scan on consecutive days. To perform ^18^F-FCH imaging, the animals were injected with approximately 5.5MBq of ^18^F-FCH intravenously and conscious uptake of the radiotracers was allowed for 30 min before initiating micro-PET imaging with the R4 microPET scanner (Concordes Microsystems Inc.). The animals were then left to recover and to allow complete the decay of radioactivity. Prior to ^18^F-FDG micro-PET imaging, the mice were fasted overnight, with water being available. On the day of ^18^F -FDG imaging, the mice were pre-warmed to a body temperature of 37°C before an approximate dose of 5.5MBq of ^18^F-FDG (0.6mM) was administered intravenously. The body temperature was maintained at 37°C with heating pad throughout the uptake period. Micro-PET imaging was performed 45 min after ^18^F-FDG injections. The duration of both ^18^F-FCH and ^18^F-FDG-PET imaging was 10 min for each mouse. During the micro-PET imaging, the mice were placed in an imaging chamber and kept under 2% isoflurane anesthesia. Both ^18^F-FDG and ^18^F-FCH were obtained from the Department of Nuclear Medicine, Singapore General Hospital.

### Micro-PET image reconstruction and analysis

Images were reconstructed by using *3-D reprojection reconstruction (3DRP)* without scatter or attenuation correction. A CT scan (described below) was obtained as a reference to determine the brain regions in the ^18^F-FDG and ^18^F-FCH PET images. Since the PET image with ^18^F-FCH can deliver an improved tumor delineation, a tumor ROI was drawn manually on the ^18^F-FCH image first. The ^18^F-FDG and ^18^F-FCH images were then merged, and the tumor ROI from the ^18^F-FCH was transferred onto the merged images to obtain the finial tumor ^18^F-FDG counts by subtracting the ^18^F-FCH counts from the merged images. The tumor to background ratio, which is defined as ^18^F-FDG counts per pixel within the tumor compared to ^18^F-FDG counts per pixel in the normal brain area, was used for further analysis. No other scoring system or interpretation criteria of metabolic activity was used in this study.

### Micro-CT procedure

A Rigaku micro-CT scanner (JMorita Engineering Co., Ltd, Japan) was adopted in the CT imaging. This *in vivo* Micro X-ray CT system consists of an X-ray tube (X-ray energy, max 90 kV), a semiconductor detector with 200 × 200 μm pixel size (acceptance surface size, 124.8 × 124.8 mm), an image reconstruction part, an optical system arm, a 3-axis sample stage part. The detector and X-ray source rotated around a fixed bed, allowing the mice to be kept in the same horizontal position in the CT scanner as in the PET scanner. The mice were transported and positioned in the CT scanner right after the PET scan. The X-ray voltage and anode current were set at 90 kVp and 50 μA, respectively. Images of the whole animal were obtained after one revolution that lasted for 17 s. No X-ray contrast medium was used.

### Bioluminescence imaging (BLI)

Anesthesia was induced with 4% isoflurane and maintained at 2% isoflurane. Mice were injected intraperitoneally with 150 mg/kg of D-luciferin potassium salt (Caliper Life Sciences), and were then incubated for 10 min to allow the distribution of D-luciferin. An integration time of 30 s was used for luminescent image acquisition by using the Xenogen Spectrum optical imaging system (Xenogen Corp., Alameda, CA). Tumor ROIs were drawn and quantified based on the Living Image software version 2.5. Specific signal was reported as the ratio of the bioluminescence signal in the ROI over the bioluminescence signal in a background region without tumor.

### Histology and immunohistochemical staining

The brain tissues were harvested 35–74 days after tumor cell inoculation. Later the tissues were stained as follows. Briefly, tissues were fixed in 4% buffered formaldehyde for 24 h, processed in paraffin, and stained with hematoxylin and eosin for histopathological examination. For immunoreactivity staining, 5 μm deparaffinized polysin-coated sections were treated with 3% hydrogen peroxide for getting rid of the endogenous oxides. A mouse anti-human Ki-67 antibody (Dako, Glostrup, Denmark) was employed for immunohistochemical staining using the peroxidase-antiperoxidase technique.

### Statistical analysis

Results of tumor-to-normal brain uptake ratios were presented as mean ± SD of the mean. The significance of differences in the uptake ratio between normal control animals and glioblastoma-bearing mice was analyzed by unpaired 2-sided Student’s *t* test.

### Method for sacrificing experimental animals

CO_2_ inhalation followed by cervical dislocation was used as the method of euthanasia at the end of animal study.

## Results

### ^18^F-FCH PET provides better contrast between tumor and normal tissues but with high false positive

**Fig 1:**
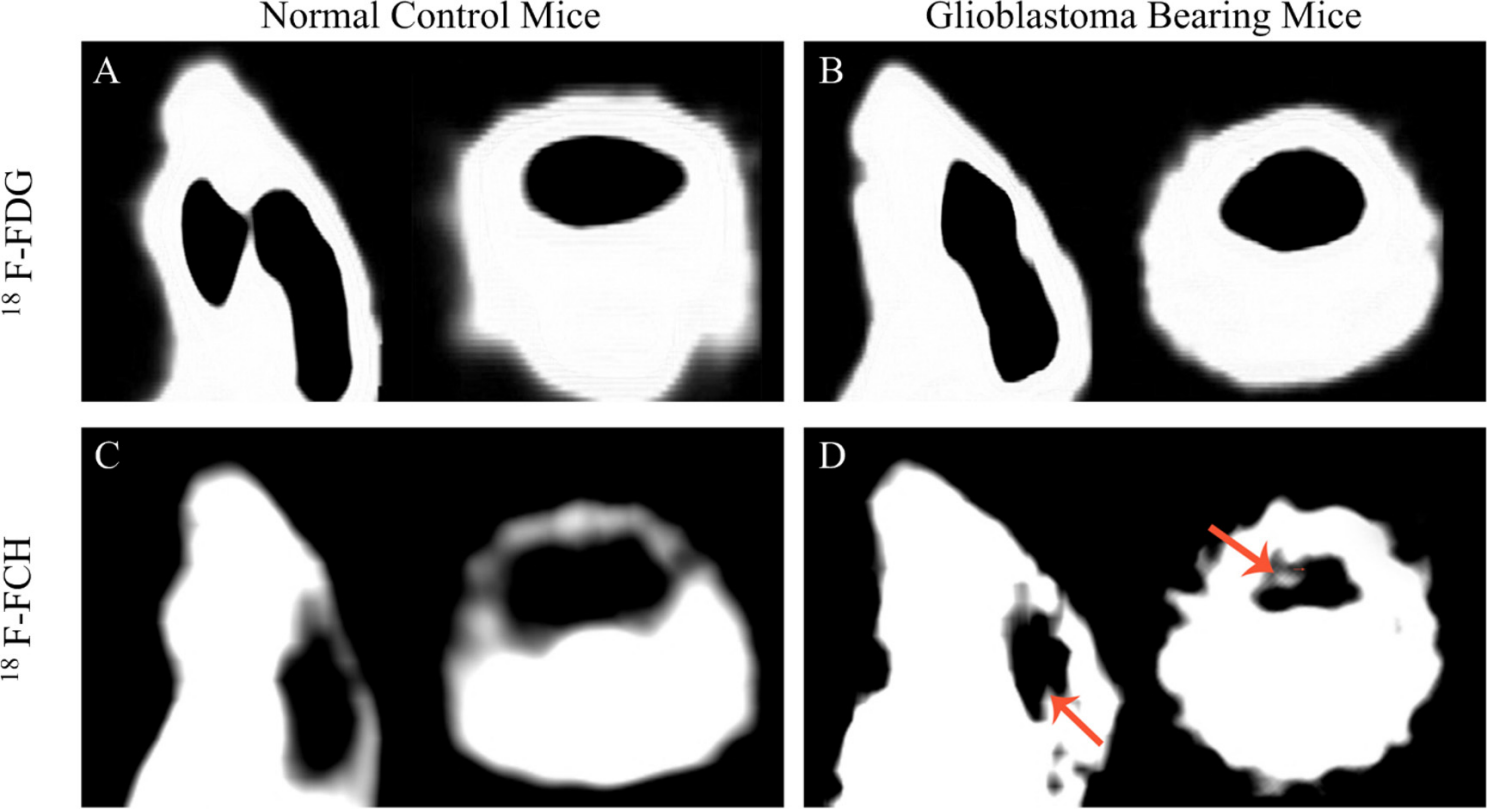
Comparison of PET images of normal control mice and glioblastoma bearing mice with different tracers. A and C: normal control mice, B and D glioblastoma bearing mice; A and B: with ^18^F-FDG, C and D: with ^18^F-FCH. The low grade glioma is illustrated by arrows.

**Fig 2:**
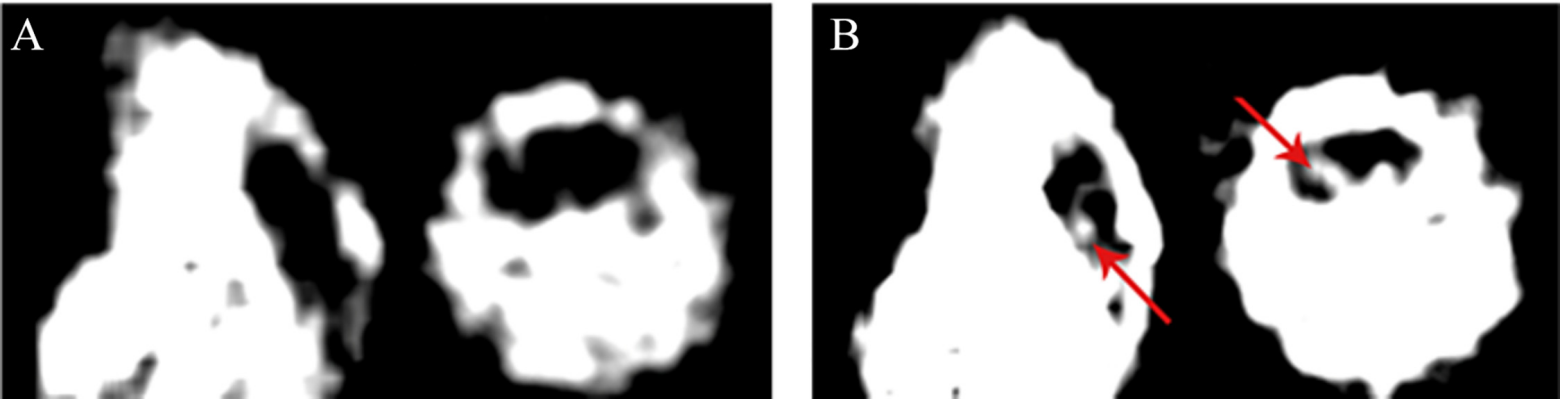
Representative ^18^F-FCH uptake in the brain of normal control mice without low grade glioma. The brain region with high ^18^F-FCH concentration is pointed by arrows. (A) No or trivial uptake of ^18^F-FCH in normal brain, (B) high concentration of ^18^F-FCH in normal brain.

Glioblastoma bearing mice and normal control mice were subjected to PET scan with single tracer administrated at one time, the images of two perpendicular cross-sections are compared in Fig 1. As expected, no tumor region can be read on the images of normal control mice with either of the tracers injected (Fig 1A and 1C). However, the image with tracer ^18^F-FDG fails to show the tumor region (Fig 1B). Only ^18^F-FCH provides a clear contrast between tumor and normal cerebral tissues (Fig 1D). It is observed that normal cerebral tissues typically represent low ^18^F-FCH uptake [12] as shown in Fig 2A. However, in some of the control animals, ^18^F-FCH uptake is unexpectedly observed in normal brain tissue (Fig 2B), resulting in potential false-positive findings. In total, 4 false positives out of 14 normal mice experiments are observed in this study, indicating a false positive rate of 28.6%. Therefore, although ^18^F-FCH can delineate the glioma clearly, the high false positive rate renders it unsuitable as a glioma imaging radiotracer.

### Combined ^18^F-FCH/^18^F-FDG-PET for imaging of glioma xenograft

**Fig 3:**
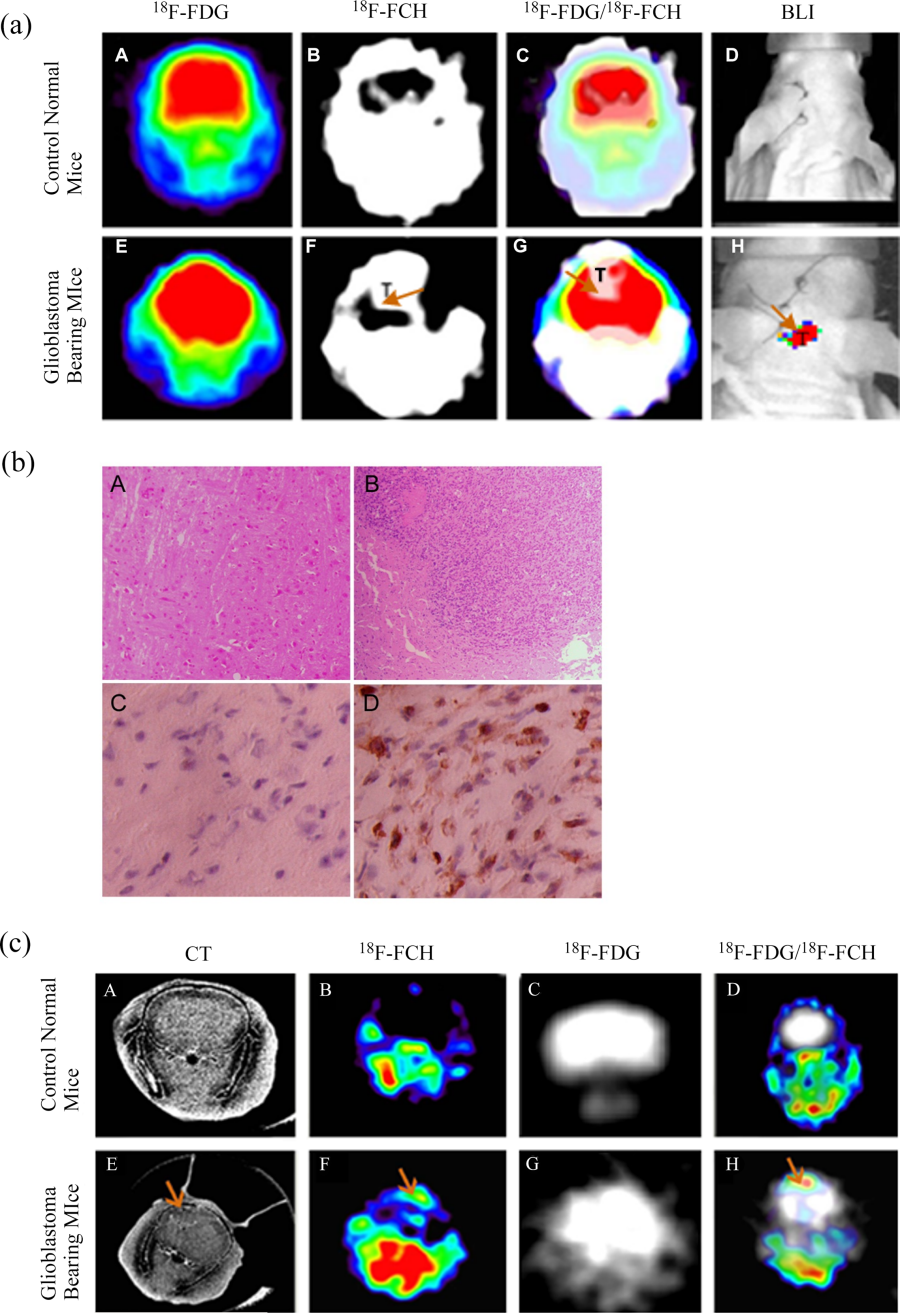
Detection of glioblastoma xenograft with dual-tracer PET confirmed by BLI, histological examination and micro CT. **(a)** PET and BLI images of a representative normal mice (A-D) and glioblastoma-bearing mice (E-H). ‘T’ denotes tumor ROIs which is further indicated by arrows. A close look of the tumor on CT image is given in E-1. **(b)** Morphological features of normal brain (A and C) and glioblastoma (B and D). Shown are representative histological sections stained with haematoxylin and eosin (100×, A and B) and Ki-67 expression (400×, C and D). **(c)** Representative microCT and/or PET images of a normal (A-D) and glioblastoma-bearing mouse (E-H). A and E: CT images without contrast agent; B and F: ^18^F-FCH PET images; C and G: ^18^F-FDG PET images; D and H: Merged images of ^18^F-FDG (in white) and ^18^F-FCH. Arrows indicate presence of tumors.

In order to detect the low grade glioma, the ^18^F-FCH and ^18^F-FDG-PET images are merged first and ROIs guided by the region with high ^18^F-FCH concentration are then manually drawn on the ^18^F-FDG images. This process is specified in Fig 3(A). As shown in Fig 3(A) A-D, no tumor can be observed in the images of normal control mice with either of the imaging techniques applied. Although ^18^F-FDG image fails to represent the tumor, the brain region can be clearly sketched in Fig 3(A) E. On the contrary, the tumor region is shown in Fig 3(A) F where ^18^F-FCH is applied as the tracer. However, the brain region is blur, resulting in the difficulty to identify the tumor boundary. The low grade glioma can only be clear observed by merging E and F, as shown in Fig 3(A) G. The result of tumor detection is also proved by the BLI image of Fig 3(A) H.

The target to background ratio (Mean ± SD) from the ^18^F-FCH and ^18^F-FDG images for each of the 4 non-tumor bearing control mice (4 false positive) and 10 tumor-bearing mice are summarized in Table 1. It is difficult to differentiate the U87 xenografts from normal brain tissues by using ^18^F-FDG only [13]. However, the tumor area delineated by ^18^F-FCH shows an increased target to background ratio, ranging from the minimum of 1.12 ± 0.02 to the maximum of 2.35 ± 0.06, with a group mean of 1.47 ± 0.37. Results show that there are 4 false positives predicted by the ^18^F-FCH images. As an improvement, all the tumor can be successfully detected by the merged ^18^F-FCH/^18^F-FDG images. The target to background ratio of the false positive area ranges from 0.98 ± 0.01 to 1.14 ± 0.07, with a group mean of 1.04 ± 0.07. This is significantly lower than the tumor to background ratio described in the non-false positive cases (*P* < 0.01).

**Table 1:** Comparison of the uptake ratio for ^18^F -FCH and ^8^F-FCH/^18^F-FDG fusion image in control and tumor-bearing mice.

Mouse	“Hot spots”/Normal cerebral uptake ratio			BLI signal ratio (tumor/background)
	^18^F-FDG	^18^F-FCH	^18^F-FDG guided by ^18^F-FCH/^18^F-FDG fusion image	
Control				
1	Unable to differentiate	2.05±0.10	1.03±0.06	
2	Unable to differentiate	1.25±0.06	0.99±0.00	
3	Unable to differentiate	1.26±0.10	0.98±0.01	
4	Unable to differentiate	2.45±0.60	1.14±0.07	
Mean±SD		1.75±0.60	1.04±0.07	
Tumor beading				
1	Unable to differentiate	2.24±0.11	1.63±0.04	15.17
2	Unable to differentiate	4.06±0.32	1.47±0.07	12.53
3	Unable to differentiate	2.37±0.39	1.36±0.05	11.01
4	Unable to differentiate	2.62±0.33	1.14±0.06	16.52
5	Unable to differentiate	4.24±0.85	1.29±0.04	24.52
6	Unable to differentiate	2.51±0.43	1.28±0.01	12.20
7	Unable to differentiate	3.31±0.38	1.12±0.02	6.26
8	Unable to differentiate	2.21±0.76	1.27±0.17	22.63
9	Unable to differentiate	1.08±0.34	2.35±0.06	89.66
10	Unable to differentiate	1.82±0.09	1.79±0.16	61.91
Mean±SD		2.65±0.98	1.47±0.37*	

*P < 0.01 for comparison with control mice (as determined by unpaired t test).

Regions of interest for calculations of uptake ratio of ^18^F -FDG in normal mice were drawn on the corresponding “hotspots” in ^18^F -FCH images. Mice with tumors showed higher ^18^F -FDG uptake ratio on fusion images with a comparable BLI signal ratio.

The detection of low grade glioma with small size is shown in Fig 3(C). As shown in Fig 3(E), the tumor is difficult to be observed based on the CT image owing to its small dimension. However, the tumor can be clearly identified in the tumor bearing mice after subtracting the ^18^F-FCH counts from the merged ^18^F-FCH/^18^F-FDG image, as shown in Fig 3(C) F-H. The same experiments were carried out on normal control mice, and results are represented in Fig 3(C) A-D, with no tumor can be observed.

### Bioluminescence imaging, histology and micro-CT

Both control and tumor-bearing mice were subjected to bioluminescence imaging (Fig 3A), but only tumor-bearing mice showed positive readouts. The growth of the xenograft is confirmed by necropsy and with standard histology techniques (Fig 3B). The tumor–bearing group shows glioblastoma xenograft on H&E–stained slices and marks by proliferation marker (Ki-67 positivity). The xenograft can be defined in CT images (the diameter of the tumor ranged from 2.8 to 4.2 mm) and corresponds well to the ^18^F-FCH PET image (Fig 3C).

**Fig 4:**
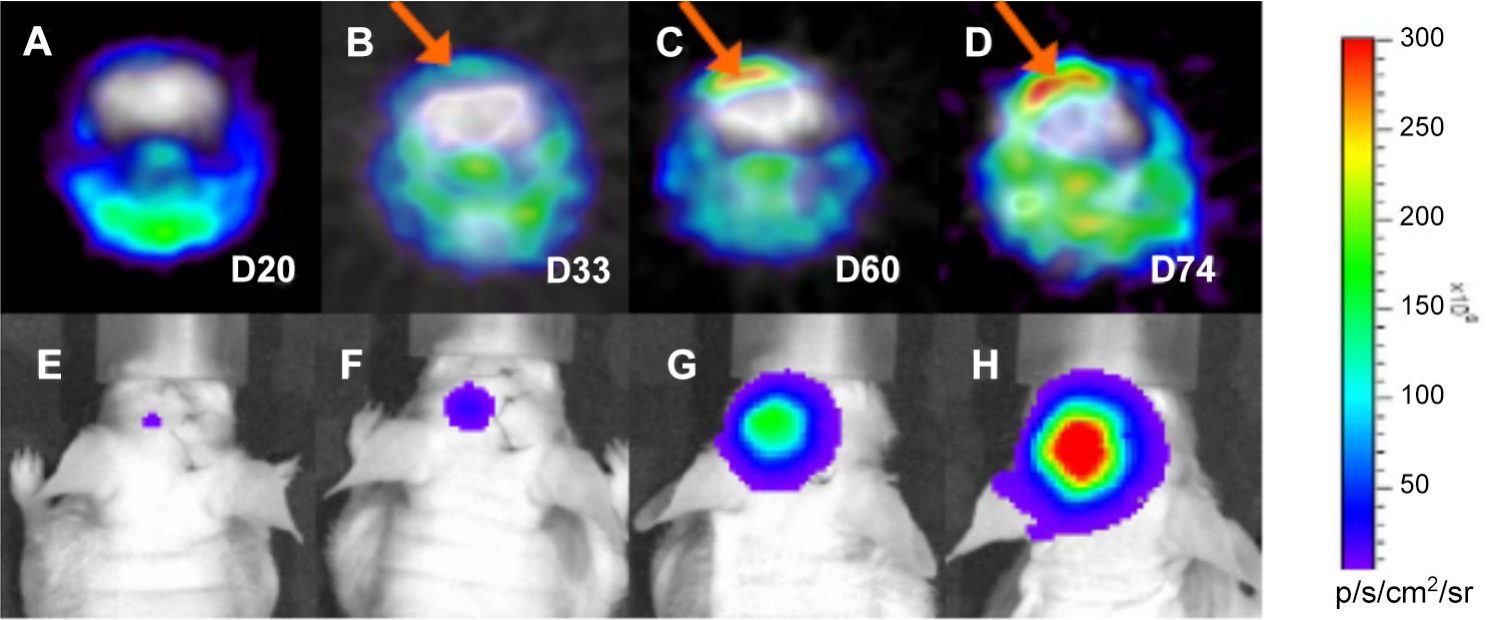
Dual-tracer PET (A-D) and BLI (E-H) monitoring growth of glioblastoma xenograft after U87-MG-luc2 cells inoculation. The growth was monitored at 20, 33, 60 and 74 days after the inoculation. Tumor ROIs are indicated by arrows in PET fusion images (B, C and D) and show increase in tracer uptake from day 33 to 74. Implanted luciferase expressing U87 MG-luc2 cells are illustrated in the brain of mouse (E-H) with a red–blue color bar as a reference of BLI signal intensity, red color indicating the highest BLI signal intensity. Increased BLI signal intensity is marked from day 20 to 74, indicating progressive growth of glioblastoma xenograft.

This technique can also be applied to monitor progression of tumor. Fig 4 illustrates the growth of glioblastoma xenograft at 20, 33, 60 and 74 days after inoculation of U87 MG-luc2 cells in a nude mouse. Increased radioactivity can be observed in the tumor from day 33 to 74 after inoculation. This is confirmed by a marked increase of BLI signal intensity from day 20 to 74.

**Fig 5:**
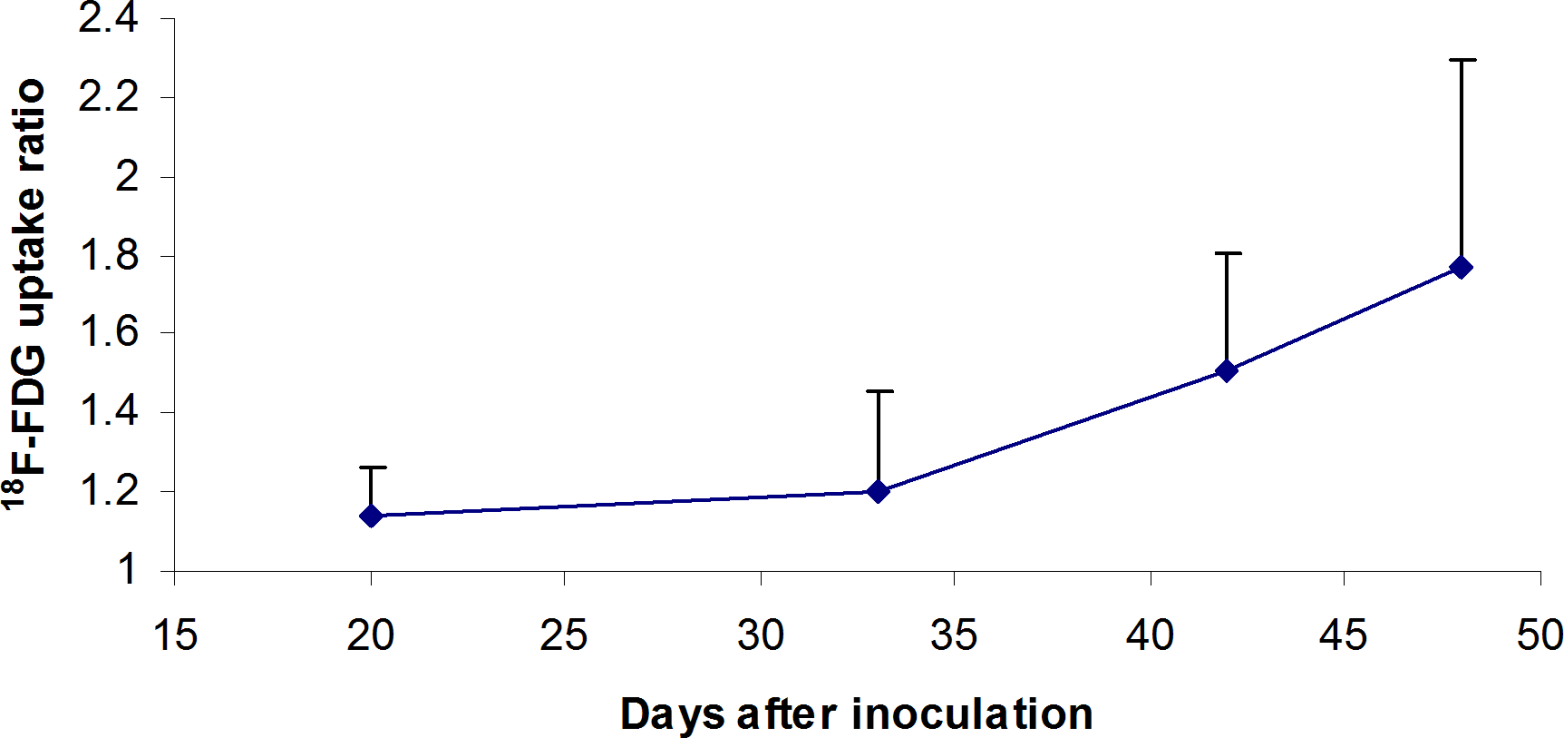
Metabolic activity of tumor measured as ^18^F-FDG uptake in tumor-to-background ratio by dual tracer technique. The graph indicates a significant increase in metabolic activity of U87 MG-luc2 cells in nude mice from day 35 to day 50 after inoculation measured by dual tracer technique (N = 3 to 6 per time point, error bars represent standard deviation).

Using this dual tracer technique, we also monitored tumor metabolic activity quantitatively in terms of ^18^F-FDG uptake ratio between tumor and background. As indicated in Fig 5, the metabolic activity of the tumor increased significantly from day 35 to day 50 after inoculation in nude mice.

## Discussion

^18^F-FDG is by far the most commonly used PET radiopharmaceutical in cancer imaging. Chemically, it is a glucose analogue and the positron emitting radioactive isotope ^18^F substitutes the hydroxyl group at the 2’ position of the glucose molecule. As a glucose analog, ^18^F-FDG is taken up by cells that significantly consume glucose such as brain, kidney and cancer cells. Once being up-taken, glucose and ^18^F-FDG undergo phosphorylation at the 6 position carbon. Unlike glucose, ^18^F-FDG will not undergo further glycolysis because the 2' hydroxyl group has been replaced by an ^18^F atom. Nevertheless, ^18^F-FDG distribution is a good reflection of the distribution of glucose uptake and tumor metabolism. Cancer cells, in order to support its fast metabolism and energy demand, require a much higher intake of energy source such as glucose. ^18^F-FDG, therefore, localizes in cancer cells. However, other cells with fast metabolism such as brain cells also take up comparative amount of glucose and ^18^F-FDG. As a result, it is difficult to differentiate low grade gliomas from surrounding normal brain cells based on ^18^F-FDG uptake only [14–16].

^18^F-FDG is primarily applied in monitoring effects of various glioma treatment modalities to determine the prognosis in patients with these lesions and to differentiate recurrent tumor due to therapy. However, its application as a first line diagnosis is limited [17–19]. Our results in this study support such findings.

^18^F-FCH is a developed PET tracer for the imaging of prostate cancer. *In vitro* and *in vivo* studies have provided substantial evidences that increased choline metabolism is a prominent feature of many cancers. Choline, a precursor for phospholipids, is transported into mammalian cells by a low affinity sodium-independent transport system and then phosphorylated by choline kinase. It is further metabolized to phosphatidylcholine, which is a primary component of cell membrane. Tumor cells usually demonstrate an elevated choline uptake compared to normal cells, which can be explained by the upregulation of choline kinase due to an increased demand of membrane constituents [20, 21].

Carbon-11 labeled Choline has been successfully used in glioma imaging, but the short half-life (20 min) of ^11^C limits its applications in clinic [22–24]. The ^18^F with a longer half-life of 120 min is able to overcome this shortcoming. As an avid substrate for choline kinase, ^18^F-FCH also undergoes phosphorylation to produce ^18^F-phosphorylfluorocholine. In cultured PC-3 (human prostate cancer) cells incubated with ^18^F-FCH, most of the intracellular ^18^F radioactivity is accounted for by the production of ^18^F-phosphorylfluorocholine [5]. Clinical data suggested that ^18^F-FCH is favorable in the imaging of many kinds of cancers, including primary brain tumors and prostate cancer that are ineffectively imaged with ^18^F-FDG [24–26].

In addition to ^18^F-FCH, there are other PET tracers which may have potential applications in glioma imaging, particularly ^18^F-labelled amino acid such as ^18^F Fluoroethyltyrosine (^18^F-FET) [27, 28]. A recent animal study has compared several potential PET tracers including ^18^F-FCH, ^18^F-FET and ^18^F-FDG for glioma imaging. Although ^18^F-FDG and ^18^F-FET have higher uptake in glioma than ^18^F-FCH, they also represent higher uptake in collateral normal cerebral tissues. The radioactivity ratio between glioma and normal tissue was 3.77 for ^18^F-FCH, 2.58 for ^18^F-FET and 1.98 for ^18^F-FDG [10]. Hence ^18^F-FCH was chosen for this study.

^18^F-FCH was applied to clinical brain tumor imaging by DeGrado *et al*. for a patient with biopsy-proven recurrent anaplastic astrocytoma [6]. Their findings were consistent with the results obtained in this study, in which a high tumor-to-normal cortex ratio was achieved. Results also showed that physiological uptake of ^18^F-FCH did occur in the pituitary gland and choroids plexus. This could possibly account for the false-positive results found in our normal control mice, as shown in Fig 2. It is worth noting that no such false positive being observed in Ref. [12] based on patient-specific studies. This difference may be caused by the *in vivo* environment of each patient, the location, growth stage and dimension of the tumor, and PET scanner, etc. Further investigations on the pharmacokinetics and pharmacodynamics of ^18^F-FCH are needed to understand the mechanism of ^18^F-FCH concentrating in normal brain cells.

Dual tracer technique has been successfully applied in the imaging of other cancers [29, 30]. One example is the imaging of parathyroid cancers in which patients are injected with ^99m^Tc-Sestamibi, which localizes in both the thyroid and parathyroid, followed by administration with ^99m^TcO4 which localizes in thyroid only. Combining the ^99m^Tc-Sestamibi and ^99m^TcO4 scan followed by subtraction of the thyroid radioactivity would enhance the sensitivity of visualization of the parathyroid cancer. However, such technique has not been applied to PET imaging. An advantage of PET over SPECT is the ability to perform quantitative imaging. As such, dual imaging technique may be even more accurate in PET than in SPECT.

We hence investigated on the potential application of a dual tracer technique in the early diagnosis of low grade glioma. The basic principle in this study is that the ^18^F-FDG concentrates in both the normal brain cells and glioma cells, making it difficult to differentiate tumor from normal tissue, particularly in low-grade glioma diagnosis. On the other hand, ^18^F-FCH would delineate the malignant area. The overlapping of the ^18^F-FDG and ^18^F-FCH images can provide an improved delineation to identify the tumor regions, and therefore develop the application of PET imaging in glioma diagnosis. A recent simulation study also confirmed dual tracer technique is superior to the single tracer in glioma PET imaging [31].

Our results demonstrated that such a dual tracer PET imaging protocol could be effective to avoid false- positive findings in ^18^F-FCH PET scan as well as identification of glioma. Guided by ^18^F-FCH delineating “hotspot” on merged images (Fig 3A) and then followed by subtraction of the ^18^F-FCH counts, all the xenografts of tumor bearing mice were successfully identified in this study, indicating a high sensitivity in diagnosis of low grade glioma.

Quantitatively, there is also a significant difference in target / background ratio between the ^18^F-FCH false- positive image and the merged ^18^F-FDG images (P<0.01). The tumor detected by the dual-tracer PET protocol is confirmed by histology (Fig 3B) and micro-CT (Fig 3C).

There are other tracers showing promise in imaging cancers such as ^18^F-fluoro-levo-thymidine (^18^F-FLT). It would be interesting to have further investigations to verify the possible application of dual tracer of ^18^F-FLT/^18^F-FDG in glioma diagnosis.

Although this study provide insights into the application of a dual tracer PET imaging technique in the low grade glioma diagnosis, there are still limitations for this technique. The availability of ^18^F-FCH is still limited to larger medical institutions with expertise in radiochemistry. Our experience with the synthesis of ^18^F-FCH is that it will take 60 to 90 minutes to synthesize ^18^F-FCH and the yield is around 8 to 15% only. Also, the additional cost, logistics and radiation adsorption to the patient due to an extra PET scan may be substantial.

## Conclusions

The potential application of a dual tracer technique for PET imaging in low grade glioma diagnosis is investigated based on a mouse orthotopic xenograft model. Results show that the dual tracer of ^18^F-FCH/^18^F-FDG with PET scan is able to improve the diagnosis of low grade glioma. By using ^18^F-FCH to clearly locate and delineate brain tumors, it is possible to effectively identify and quantify the metabolic activity of the brain tumors on the merged ^18^F-FCH/^18^F-FDG PET images. Such findings might have great impact on clinical studies for early detection of low grade glioma. Further investigations are warranted to evaluate potential of this technique in clinical settings.

## Supporting Information

S1 ARRIVE Checklist ARRIVE Guidelines Checklist Animal Research: Reporting In Vivo Experiments. Title: Page 1.Abstract: Page 3.Background: Pages 4–5. IntroductionObjectives: Page 5.Ethical statement: Page 5. Section 2.Study design: Pages 5–8. Section 2.Experimental procedures: Pages 5–8. Section 2.Experimental animals: Pages 5–6. Sections 2.1 & 2.2.Housing and husbandry: Page 5. Section 2.1.Sample size: Page 5. Section 2.1.Allocating animals to experimental groups: Page 8. Section 2.8.Experimental outcomes. Pages 8–11. Section 3.Statistical methods: Page 8. Section 2.8.Baseline data: Page 8. Section 3.1.Numbers analyzed: Pages 8–11. Section 3.Outcomes and estimation: Page 8. Section 2.8.Adverse event: Not applicable.Interpretation/ Scientific implications: Pages 11–14. Section 4.Generalization / Translation: N/A.Funding: Page 15. Acknowledgements.
(PDF)
